# Optical optimization of double-side-textured monolithic perovskite–silicon tandem solar cells for improved light management

**DOI:** 10.1039/d0ra04634e

**Published:** 2020-07-16

**Authors:** Fazal E. Subhan, Aimal Daud Khan, Adnan Daud Khan, Najeeb Ullah, Muhammad Imran, Muhammad Noman

**Affiliations:** US-Pakistan Center for Advanced Studies in Energy, University of Engineering & Technology Peshawar 25000 Pakistan; Arizona State University Tempe Arizona 85287 USA fsubhan@asu.edu; College of Energy, Soochow Institute for Energy and Materials Innovations (SIEMIS), Soochow University Suzhou 215006 China; Key Laboratory of Advanced Carbon Materials and Wearable Energy Technology of Jiangsu Province, Key Laboratory of Modern Optical Technologies of Ministry of Education Suzhou 215006 China; Department of Electrical Engineering, Military College of Signals, National University of Sciences and Technology (NUST) Islamabad 46000 Pakistan

## Abstract

Tandem configuration-containing perovskite and silicon solar cells are promising candidates for realizing a high power conversion efficiency of 30% at reasonable costs. Silicon solar cells with planar front surfaces used in tandem devices cause high optical losses, which significantly affects their efficiency. Moreover, some studies have explored the fabrication of perovskites on textured silicon cells. However, due to improper texturing, light trapping is not ideal in these devices, which reduces the efficiency. In this work, we optimized the pyramid height of textured silicon cells and efficiently characterized them to achieve enhanced light trapping. Two different kinds of perovskites, namely, Cs_0.17_FA_0.6_Pb(Br_0.17_I_0.7_)_3_ and Cs_0.25_FA_0.6_Pb(Br_0.20_I_0.7_)_3_ with wide bandgaps were conformally deposited on textured silicon cells, and the performance of these flat and fully textured tandem devices was numerically analyzed. The thickness of each layer in the tandem cell was optimized in a way to ensure a perfect current match between the top perovskite and bottom silicon subcells. The results indicated that the textured tandem configuration enhances light absorption over a broad spectral range due to the optimized pyramid height compared to flat surfaces. Eventually, the photovoltaic parameters of the proposed tandem cell were compared with the already existing structures, and our design supports large values of open circuit voltage (*V*_oc_) = 1.78 V, short circuit current density (*J*_sc_) = 20.09 mA cm^−2^, fill factor (FF) = 79.01%, and efficiency (*η*) = 28.20% compared to other kinds of tandem solar cells.

## Abstract 2

Tandem configuration-containing perovskite and silicon solar cells are promising candidates for realizing a high power conversion efficiency of 30% at reasonable costs.

## Graphical abstract

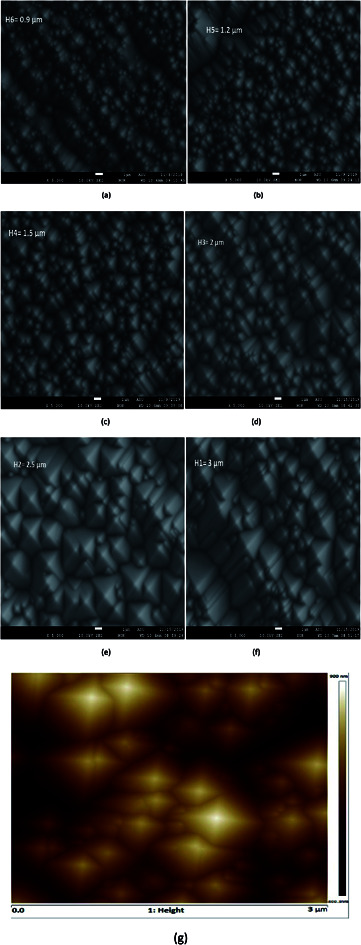

## Introduction

The crystalline silicon (c-Si) technology has dominated the photovoltaic market with continuously improving efficiency and cost-effectiveness.^1^ Due to intensive research, the current efficiency of c-Si-based solar cells reached approximately 26.1%,^2,3^ which is close to their theoretical Auger efficiency limit of 29.4%.^4^

Selecting a top cell partner with a wide band gap to reduce thermal losses and enhance solar spectral utilization is an effective way to achieve an efficiency value beyond the Auger efficiency limit for silicon solar cells. In recent times, a dual junction combining III–V materials and Si reached a power conversion efficiency (PCE) of 32.8%.^5^ However, due to expensive deposition techniques, III–V material-based solar cells have not yet attracted attention in the PV market. Recently, organic–inorganic metal halide perovskites such as cesium–formamidinium-based Cs_*y*_FA_1−*y*_Pb(Br_*x*_I_1−*x*_)_3_ with wide bandgaps of 1.63 eV and 1.68 eV have been recognized as unique light-absorbing materials that have passed the record efficiency.^6,7^ The perovskite material is found to be the best partner for Si solar cells due to their long carrier diffusion lengths, sharp optical absorption edge, low exciton binding energies, and excellent defect tolerance.^8–12^ These characteristics theoretically allow the perovskite/silicon tandem solar cells to grasp efficiencies above 30% at reasonable production costs.^13,14^ Experimentally, power conversion efficiencies over 26% and 27% have been achieved for perovskite–silicon monolithic two-terminal and mechanically stacked four-terminal arrangements, respectively.^15–17^

The two-terminal perovskite–silicon tandem cell has achieved greater attention due to extensive use in industries with the least number of processing steps, substrates, and interconnection requirements.^18^ Some research groups have investigated both cells with planar front surfaces^19^ and those with textured surfaces.^20^ The textured silicon solar cell is found to be better due to efficient light trapping in both silicon and top subcell perovskites. The textured solar cell with randomly placed pyramids of various size distributions (i) helps in the reduction of reflectance by a flat surface from over 30% to less than 10%, (ii) leads to enhanced absorption nearer the forward-facing collecting junction, with the inclined ray tracing inside the absorbing layer, and (iii) contributes to efficient light trapping *via* total internal reflection on the surface owing to the geometric texture.^21^ It is noteworthy to mention that the reflectance is high for randomly placed pyramids with low size distribution, while pyramids with large heights of typically 3–10 μm size distribution hinder the subsequent processing stages and the conformal deposition of perovskites.^22,23^ R. Pandey *et al.* have numerically demonstrated the hysteresis of moisture-free tandem perovskite–Si solar cells with flat surfaces. However, due to poor light trapping, they were able to achieve an efficiency of only 23.1%.^24^ K. A. Bush *et al.* have fabricated a 23.6% efficient single-side-textured perovskite–silicon tandem cell with the front side polished.^25^ However, due to reflection losses from the top surface, they did not succeed in attaining high efficiencies. E. Köhnen *et al.* have fine-tuned the perovskite absorber and the indium zinc oxide (IZO) flat front electrode to achieve photocurrents well above 19 mA cm^−2^ with a stabilized efficiency of 26.0%.^26^ However, they did not consider double-side texturing for improved light trapping to achieve high matched short-circuit current for further efficiency enhancement. Thus, the polished surface leads to strong front surface reflection losses, poor match short circuit current and inefficient light trapping. Therefore, a c-Si bottom cell with a heterojunction configuration needs better light management, in particular in the infrared part of the solar spectrum, where it absorbs weakly. It has been found that by introducing double-side texturing, the current match can be potentially improved up to 2–4 mA cm^−2^.^27–29^ F. Sahli *et al.*, have demonstrated 25.2% efficient double-side-textured monolithic two-terminal perovskite/silicon tandem cells with improved optical modelling.^30^ However, the optimization of pyramid height, charge transport and transparent conductive oxide (TCO) layer thickness is still necessary for high-matched short-circuit current and conformal deposition of perovskites on textured surfaces. Recently, B. Chen *et al.*, have achieved a perovskite/silicon tandem cell with an efficiency of 26% on textured silicon.^31^ However, improvement in efficiency is still required to get close to the Auger limit.

Herein, we consider anisotropic etching to get optimized random pyramids with the 〈111〉 facets in order to improve the optical response of the cell in the near infrared region *via* a light scattering effect. Different c-Si pyramid heights and qualities of the textured c-Si surface are characterized using a scanning electron microscope (SEM) and an atomic force microscope (AFM), respectively to create a guide to maximize the current and matching in perovskite/silicon tandem cells and provide a way aimed at conformal top cell deposition. Two kinds of perovskite materials such as Cs_0.17_FA_0.6_Pb(Br_0.17_I_0.7_)_3_ and Cs_0.25_FA_0.6_Pb(Br_0.20_I_0.7_)_3_ having bandgaps of 1.63 and 1.68 eV, also referred to as Cs_17_/Br_17_ and Cs_25_/Br_20_ through their Cs and Br contents, were combined with textured silicon along with essential optimized functional layers to make efficient monolithic perovskite–silicon tandem solar cells. The proposed cells are found to exhibit good optical properties compared to flat top surfaces. Moreover, the highest efficiency of 28.20% is achieved in case of Cs_25_/Br_20_–Si tandem cells.

## Experimental work

Experimental samples were fabricated for our research using the facilities at Solar Power Laboratory (SPL), Arizona State University, USA. Monocrystalline silicon wafers can generally be etched in alkaline aqueous solutions of sodium hydroxide (NaOH), sodium carbonate (Na_2_CO_3_), inorganic potassium hydroxide (KOH) commonly called caustic potash, and tetramethylammonium hydroxide (TMAH), intended to produce a random pyramid upright on the etched surface. However, here we used only 500 mL KOH water solutions (Sigma-Aldrich) of two different KOH concentrations, namely, 35 wt%, and 45 wt%. The polished c-Si samples of 200–250 μm thickness with a resistivity in the range of 1–5 Ω cm were cut into pieces of 2 × 2 cm^2^ dimension and kept in a KOH water solution containing perfluoroalkoxy alkane (PFA) in a sample holder for different etching time periods. All the experiments were performed at 75 °C, as the temperature ranging from 60 to 80 °C has no significant effect on the feature size during sample processing. The temperatures were measured directly by dipping a Teflon-coated thermometer into the cast-off chemical solutions. The textured silicon samples of height H6, H5, and H4 were attained using 43 wt% KOH solution for etching durations of 10, 20, and 30 minutes, respectively. While samples of pyramid height H3, H2, and H1 were attained using 34 wt% KOH solution for etching durations of 10, 20, and 30 minutes, respectively. The final morphology of the facets has various inferences on the performance of silicon solar cells, which include reduced front-surface reflectance, improved light trapping in the infrared region in silicon and light scattering at a shorter wavelength, conformal top cell deposition, and reduced surface recombination.

### Sample characterization

An SEM and an AFM were used to study the surface morphology of the fabricated textured silicon sample with different pyramid heights. Furthermore, the optical response (total reflectance and transmittance spectra) of the sample was attained using a PerkinElmer Lambda 950 ultra violet-visible near infrared (UV-VISNIR) spectrophotometer equipped with an integrating sphere. The vital step was taken using a PerkinElmer Automated Reflectance/Transmittance Analyzer (ARTA) equipped with a UV-VISNIR spectrophotometer to characterize the samples that receive light from direct and non-normal viewpoints with respect to the wavelength, incident angle, and polarized-resolved transmittance and reflectance. In addition, to determine where all the incident light goes, the ARTA was used at a wavelength of 270 nm using a user-specified measurement table with a step of 40 for the sphere detector compared with respect to textured samples. The aim of a shorter wavelength of 270 nm for ARTA operation is to examine a shorter wavelength response incident on the silicon wafer using angular resolved reflectance by placing the detector film in front of the solar cell.

## Results and discussion

**Fig. 1:**
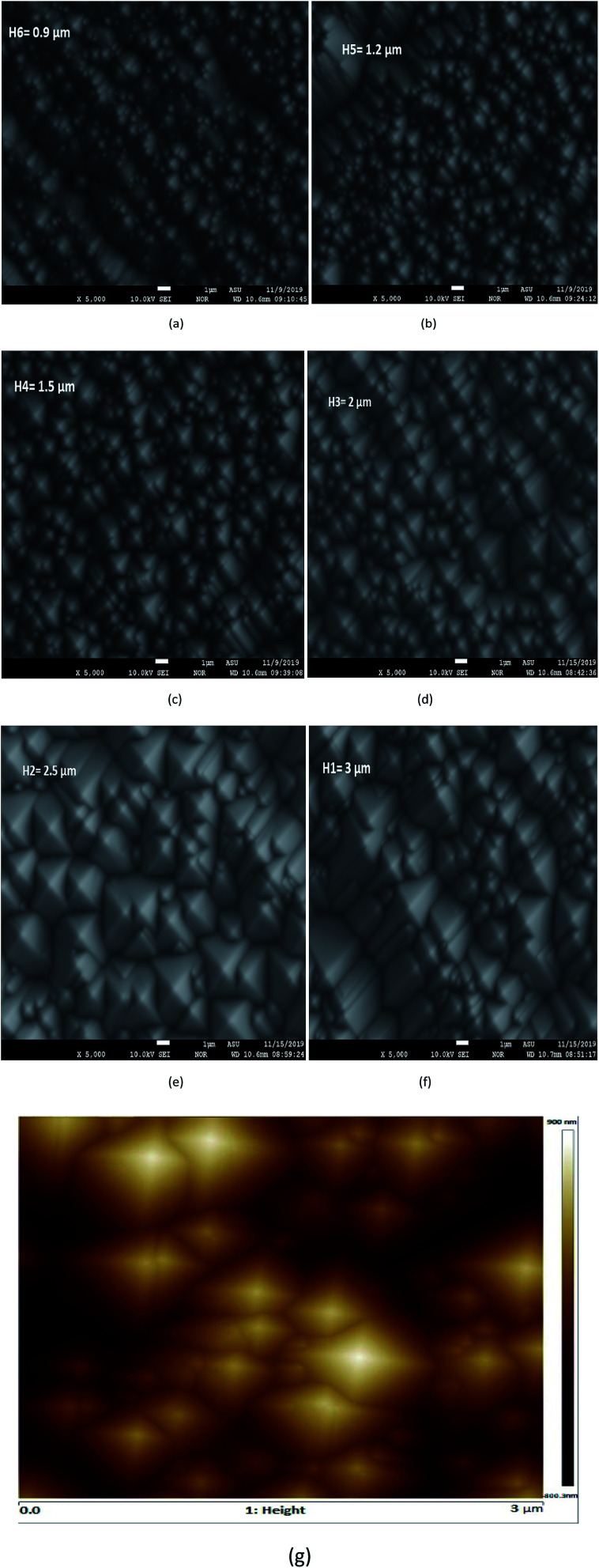
(a–f) SEM images of the textured silicon sample to analyze the surface topography of different pyramid heights under 5000 resolution, and (g) AFM image of textured silicon.

In order to understand the improvement in the photovoltaic parameters of the proposed solar cell, we started our discussion with a simple silicon sample. The pyramid type of texturing of different heights was produced on both sides of the silicon sample with the aim of optimizing the pyramid height of silicon, which is important for light trapping in the tandem configuration. Fig. 1(a)–(f) shows the SEM images of the textured silicon sample with different heights. Moreover, to perform a quantitative analysis, a round of AFM mapping by means of the Bruker multimode was performed in air with a silicon pyramidal tip in the tapping mode with 25.0 μm^2^ image projected area, as shown in Fig. 1(g). The average pyramid height of textured surfaces and corresponding base angles were calculated using the Nanoscope and Gwyddion modular post-processing software programs for SPM (scanning probe microscopy) data imagining and analysis.

**Fig. 2:**
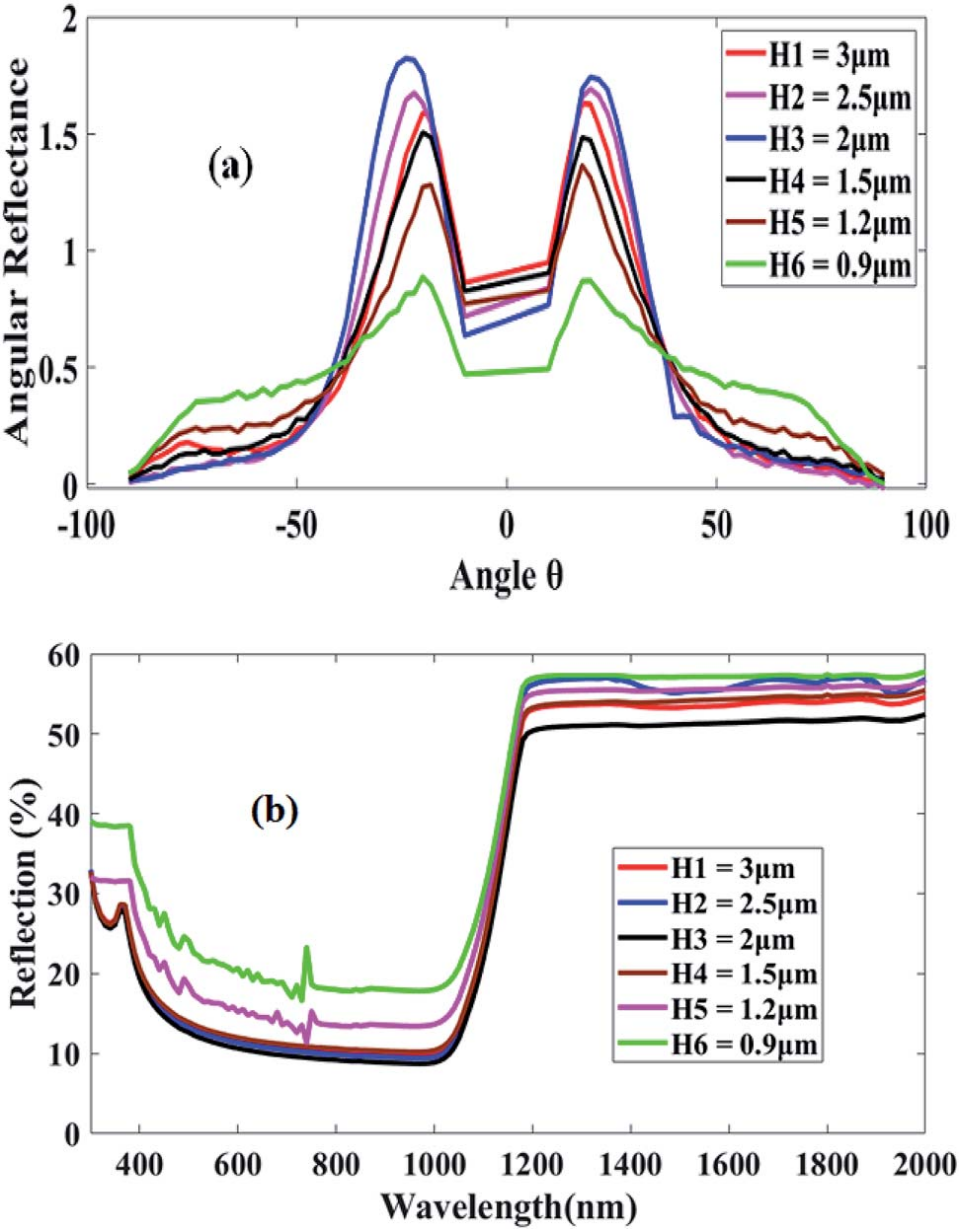
(a) Angular resolved reflectance of textured c-Si with different pyramid heights, and (b) reflectance of textured c-Si with different pyramid heights (extracted from AFM).

For evaluating the optical response of the textured silicon sample, we used an ARTA and a spectrophotometer. Fig. 2(a) shows the angular reflectance of the textured sample obtained at different pyramid heights ranging from 3 to 0.9 μm size. These ARTA results were obtained from an angle in the range of −90 to 90 degree for an entire front surface coverage. The results indicated that H1 of 3 μm was not a suitable option for solar cell applications because it supports a maximum reflection of 0.91 at −24° with a base angle of 51°. Similarly, the H2, H3 and H4 heights were not good options because they exhibited maximum reflection peaks. The height H6 appeared to provide very low reflection; however, at large angles, the reflection gradually maximized. Therefore, the height H5 of 1.2 μm was comparatively suitable in our case. Moreover, the scattering effect, which is significant for light trapping, usually comes into picture when the pyramid height is equal or closer to silicon bandgap energy.^32^ In our case, the scattering effect is only supported by heights H5 and H6 at a wavelength in the range of 400–800 nm, as shown in Fig. 2(b). Therefore, we have chosen a pyramid height between 0.9 and 1.2 μm for our further analyses of perovskite/silicon tandem development in the simulation environment.

### Optical simulations

**Fig. 3:**
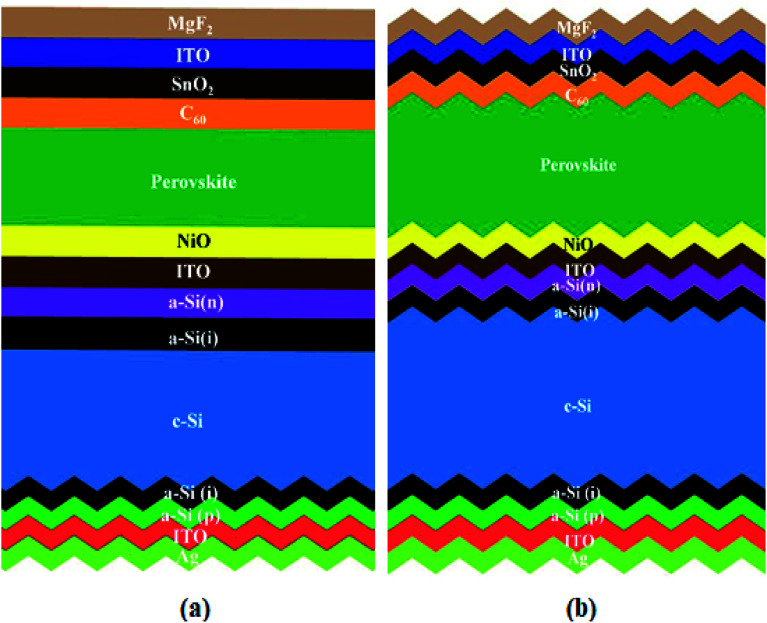
Perovskite/silicon tandem solar cells with (a) front side polished and (b) double sides textured.

We used the optimum pyramid height of the silicon sample and deposited a wide bandgap perovskite material on top of it. In this way, we made a complete perovskite/silicon tandem solar cell with all functional layers, as shown in Fig. 3. The description of each layer is as follows: MgF_2_ acted as an anti-reflective coating layer, which is responsible for minimizing the reflection losses from the top surface;^33^ indium tin oxide (ITO) was used as a transparent conductive oxide for the front contact; tin oxide (SnO_2_) was used as a buffer layer; buckminsterfullerene (C_60_) was used as an electron transport layer; the wide band gap perovskite was used as an absorber for the top subcell; nickel oxide (NiO) was used as a hole-selective layer followed by an interconnecting ITO layer; the bottom subcell contained textured silicon heterojunction (HJ), consisting of n+ doped and intrinsic (i) hydrogenated amorphous silicon (a-Si:H) layers, the textured c-Si wafer; i and p+ doped a-Si:H layers and the textured silver (Ag) back reflector and contact. The simulations are performed using the Sun Solve software from the PV lighthouse. Sun Solve is an effective tool, which gives optical information about each layer by combining Monte-Carlo-based ray tracing and thin-film optics.^34^ The optical constants for perovskites and all other layers including MgF_2_, crystalline silicon, silver, intrinsic and doped (n- and p-type) amorphous silicon (a-Si:H), the intermediate ITO layer, and the rear ITO layer were taken from the literature.^6,35–39^

As discussed, for analysis, we have considered two different kinds of wide bandgap perovskite materials such as Cs_17_/Br_17_ having a bandgap of 1.63 eV and Cs_25_/Br_20_ having a bandgap 1.67 eV, respectively. The thicknesses of each layer of both kinds of perovskites (Cs_17_/Br_17_, Cs_25_/Br_20_) in a tandem configuration were optimized, which are displayed in Table 1. The optimization is performed by matching currents between perovskite and silicon subcells. Thus, in this way, a thickness of 500 nm was obtained for Cs_17_/Br_17_ and 650 nm for Cs_25_/Br_20_, and all other thicknesses remained constant. Moreover, the short-circuit current density (*J*_sc_) was evaluated by taking integration of the product of the absorption spectrum as a function of wavelength under AM1.5G spectral irradiance over the wavelength range of 300–800 nm for Cs_17_/Br_17_, while the spectral range was 300–760 nm for Cs_25_/Br_20_. Table 2 indicates the thickness and corresponding current density values of each layer in a tandem configuration. A perfect match was obtained between the current density of perovskites and silicon subcells.

**Table 1:** Thicknesses of the functional layers of perovskite solar cells

Layer	Perovskite Cs_17_/Br_17_ thickness (nm)	Perovskite Cs_25_/Br_20_ thickness (nm)
Front ITO	120	120
SnO_2_	15	15
C_60_	15	15
Perovskite	500	650
NiO_*x*_	20	20
Rear ITO	20	20
Glass	1000	1000
Ag	300	300

**Table 2:** Thicknesses and current density values for the perovskite/silicon tandem solar cell

Layer	Perovskite/silicon tandem layer thickness (nm)	*J*_sc_ (mA cm^−2^)
MgF_2_	100	0.065
ITO front	120	1.653
SnO_2_	15	0.256
C_60_	15	1.214
Perovskite (Cs_17_/Br_17_/Cs_25_/Br_20_)	500/650	20.34
NiO_*x*_	20	0.325
Intermediate ITO	20	0.418
a-Si:H(n)	8	0.005
a-Si:H(i)	8	0.002
c-Si	250 000	20.09
a-Si:H(i)	8	0.000
a-Si:H(p)	11	0.000
Rear ITO	180	0.431
Ag	300	0.225

**Fig. 4:**
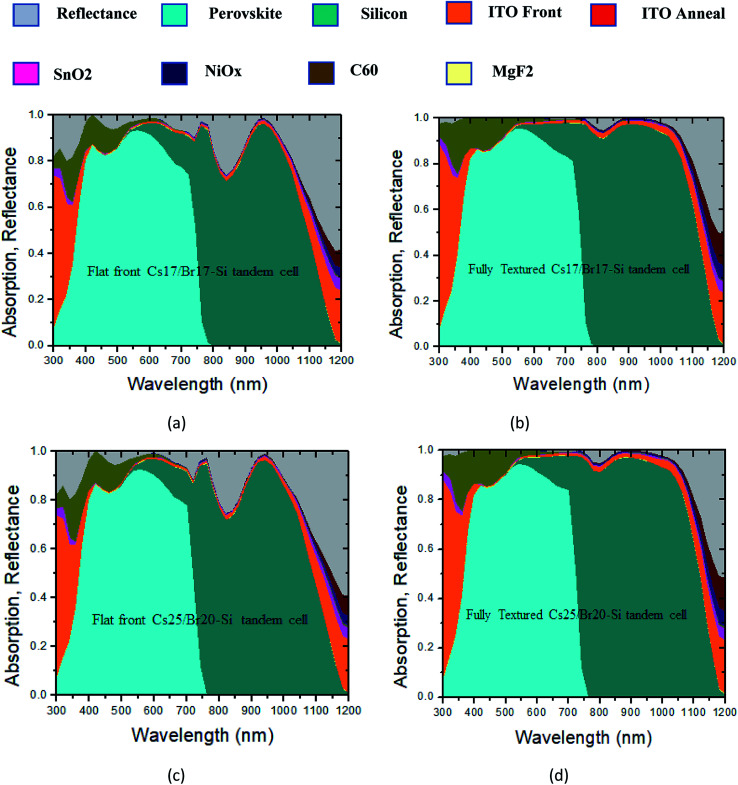
Absorption and reflectance spectra of perovskite/Si tandem solar cells with (a) front-side-polished Cs_17_/Br_17_–Si, (b) double-side-textured Cs_17_/Br_17_–Si, (c) front-side-polished Cs_25_/Br_20_–Si, and (d) double-side-textured Cs_25_/Br_20_–Si, respectively.

In order to analyze the performance of the two types of tandem cells (Cs_17_/Br_17_–Si, and Cs_25_/Br_20_–Si), we have simulated the cells with flat and textured top perovskite subcells. It has to be noted that the bottom silicon subcell is textured in both kinds of tandem cells. Fig. 4(a) shows the absorption and reflectance spectra of a Cs_17_/Br_17_–Si tandem configuration with flat perovskite top subcells. It appears that the reflectance indicated by the gray curve is relatively large at long wavelengths, which essentially reduces the current density in this spectral region. Moreover, the current mismatching was high in this case; perovskite exhibited 20.34 mA cm^−2^, while silicon supported 18.44 mA cm^−2^. This current mismatch between the cells will lead to low efficiency and eventually reduce the life time of the cell. In Fig. 4(b), the absorption and reflectance spectra were calculated for the double-side-textured surface. In this case, the absorption in the long wavelength region is significantly improved due to scattering effect induced by the textured surfaces. The difference in the current density values reduced from 1.86 mA cm^−2^ (flat perovskite) to 0.25 mA cm^−2^, which is quite acceptable in tandem configuration.^26^Fig. 4(c) shows the absorption and reflectance spectra of a Cs_25_/Br_20_–Si tandem configuration with flat perovskite top subcells. The absorption characteristics in this case slightly changed compared to the previous case. Herein, the current mismatch was 1.65 mA cm^−2^, which was somewhat lower than that of the previous case. However, by introducing double-side texturing (Fig. 4(d)), the difference in the current matching appears the same as calculated in Fig. 4(b). Furthermore, the tandem models were tested with planar and double-side-textured silicon surfaces, and it was found that the planar structure limits the photocurrent value to ∼18.44 mA cm^−2^, a value that is ∼2 mA cm^−2^ lesser in comparison to a double-side-optimized texture design. Finally, in order to show which of the tandem configurations is better, we calculated the photovoltaic parameters including short current density (*J*_sc_), open circuit voltage (*V*_oc_), fill factor (FF), and efficiency (*η*), as shown in Table 3. It becomes clear that the tandem cell of type Cs_25_/Br_20_–Si is the best option because of improved photovoltaic parameters compared to Cs_17_/Br_17_–Si. The highest conversion efficiency obtained was about 28.20%, which indicated that the proposed cell may be used for commercial applications.

**Table 3:** Photovoltaic parameters of Cs_17_/Br_17_–Si and Cs_25_/Br_20_–Si tandem solar cells

Perovskite–silicon tandem cell	*V*_oc_ (V)	*J*_sc_ (mA cm^−2^)	FF (%)	*η* (%)
Cs_17_/Br_17_–Si	1.72	20.09	77.43	26.75
Cs_25_/Br_20_–Si	1.78	20.09	79.01	28.20

We finally compared the proposed efficient tandem solar cell with the existing tandem structures, as shown in Table 4. Our cell supports relatively large values of *V*_oc_, *J*_sc_, FF, and *η*, respectively, which indicates that it can be used for practical applications.

**Table 4:** Comparison with various types of tandem solar cell structures

Reference	Structure	*V*_oc_ (V)	*J*_sc_ (mA cm^−2^)	FF (%)	*η* (%)
24	Monolithic single side textured perovskite–c-Si HJ	1.75	16.89	74.30	21.93
25	Monolithic planar perovskite–c-Si HJ	1.69	18.04	75.43	23.08
30	Monolithic fully textured perovskite–c-Si HJ	1.78	19.5	73.10	25.20
26	Monolithic planar perovskite–c-Si HJ	1.76	19.2	76.50	26.00
17	4-Terminal planar perovskite–c-Si HJ	—	—	—	27.0
31	Monolithic fully textured perovskite–c-Si HJ	1.82	19.3	74.4	26.0
Current study	Proposed monolithic perovskite–c-Si HJ	1.78	20.09	79.01	28.20

## Conclusion

In summary, an experimental investigation has been performed to optimize the pyramid height of the textured silicon cell to improve light trapping in solar cells. Two different perovskite materials such as Cs_0.17_FA_0.6_Pb(Br_0.17_I_0.7_)_3_ and Cs_0.25_FA_0.6_Pb(Br_0.20_I_0.7_)_3_ with wide bandgaps have been deposited on the optimized textured silicon cell to form a tandem solar cell with all essential functional layers. To evaluate the performance of the proposed tandem cells, a comparison has been made between flat and fully textured surfaces. In case of the flat surface tandem solar cell, the current mismatch between the top and bottom subcells was quite high (1.86 mA cm^−2^) compared to the fully textured tandem solar cell (0.25 mA cm^−2^). Moreover, improved absorption efficiencies and less reflection losses have been obtained in case of the textured tandem solar cell compared to the flat cell. Finally, the photovoltaic parameters of our cell have been compared to existing solar cells, and it has been found that our proposed cell appears to support high values of *V*_oc_ = 1.78 V, *J*_sc_ = 20.09 mA cm^−2^, FF = 79.01%, and *η* = 28.20%, which indicates that our design could be a better option for the photovoltaic community.

## Conflicts of interest

There are no conflicts of declare.

## Supplementary Material
